# Bearing Fault Diagnosis Using a Particle Swarm Optimization-Least Squares Wavelet Support Vector Machine Classifier

**DOI:** 10.3390/s20123422

**Published:** 2020-06-17

**Authors:** Mien Van, Duy Tang Hoang, Hee Jun Kang

**Affiliations:** 1 Centre for Intelligent and Autonomous Manufacturing Systems, and School of Electronics, Electrical Engineering and Computer Science, Queen’s University Belfast, Belfast BT7 1NN, UK; m.van@qub.ac.uk; 2 Department of Electrical Engineering, University of Ulsan, Ulsan 44610, Korea; hoang.duy.tang@gmail.com; 3 School of Electrical Engineering, University of Ulsan, Ulsan 44610, Korea

**Keywords:** non-local means (NLM), empirical mode decomposition (EMD), support vector machine (SVM), wavelet kernel, minimum redundancy maximum relevance (mRMR), particle swarm optimization (PSO), bearing fault diagnosis

## Abstract

Bearing is one of the key components of a rotating machine. Hence, monitoring health condition of the bearing is of paramount importace. This paper develops a novel particle swarm optimization (PSO)-least squares wavelet support vector machine (PSO-LSWSVM) classifier, which is designed based on a combination between a PSO, a least squares procedure, and a new wavelet kernel function-based support vector machine (SVM), for bearing fault diagnosis. In this work, bearing fault classification is transformed into a pattern recognition problem, which consists of three stages of data processing. Firstly, a rich information dataset is built by extracting the features from the signals, which are decomposed by the nonlocal means (NLM) and empirical mode decomposition (EMD). Secondly, a minimum-redundancy maximum-relevance (mRMR) method is employed to determine a subset of feature that can provide an optimal performance. Thirdly, a novel classifier, namely LSWSVM, is proposed with the aid of a PSO, to provide higher classification accuracy. The key innovative science of this work is to propropose a new classifier with the aid of an new wavelet kernel type to increase the classification precision of bearing fault diagnosis. The merit features of the proposed approach are demonstrated based on a benchmark bearing dataset and a comprehensive comparison procedure.

## 1. Introduction

Since bearing is a crucial component in the machine, its failure will hugely affect to the disruption of the machine. Therefore, condition monitoring for rolling bearings has become more and more important to detect early damage and increase safe of the operating systems. In the literature, two approaches can be applied to detect the bearing defects: (1) acoustic signal analysis, where the acoustic signal is acquired to obtain bearing characteristic information, and (2) vibration signal analysis, where the vibration signal is acquired. Among them, using vibration signal usually provides better defect detecting accuracy becuase it contains rich information of the bearing characteristics and less measurement noise [1].

Bearing defects can be detected by either analyzing the fault frequency spectrum [2] or pattern recognition [3]. However, the analysis in [4] shown that the pattern recognition can give higher accuracy compared to the spectrum approach. In the approach of traditional pattern recognition, the system will include three major components: feature extraction, feature selection and feature classification. The goal of the feature extraction task is to get as much information about the condition of the system as good. For this purpose, we employ the NLM-EMD method, which has been developed in our previous work [5] and proved its effectiveness, to extract a rich bearing feature set.

Feature extraction usually results in a large feature set. Unfortunately, the large feature set does not neccessarily provide higher classification accuracy as it possibly contains irrelevant and redundant features. Thus, it is signiticant to eliminate the irrelevant and redundant features before it is fed back to a classifier. To obtain an optimal feature subset, a minimum-redundancy maximum-relevance (mRMR) feature selection method has been developed [6]. The mRMR tries to search an outstanding combination of candidate features for minimum redundancy and maximum relevance. Due to the merits of the mRMR, it is employed in this paper to select the effective features.

Once the salient features are selected, they are fed into a classifier to identify the system condition. Due to its high performance classification and less requirement on sample data input, the support vector machine (SVM) proposed by Cortes and Vapnik [7] has been successfully applied to signal processing [8], regression analysis [9], pattern recognition [10], and bearing fault diagnosis [11]. However, the original SVM classifier provides high computational burden due to the method used to solve the quadratic programming problem in the SVM [12]. In order to reduce this, many methods have been developed, for example the SVM light decomposition algorithm [13], sequential minimal optimization (SMO) algorithm [14], neighbor algorithm [15], and least squares SVM (LSSVM) [16]. Among them, the LSSVM is commonly applied in real applications due to its simplicity in implementation and efficiency in classification and computation [17].

In the SVM classifier, a kernel function is used to transform the data from the lower dimension space to a high dimension space. Hence, the prior selection of the kernel will decide the way of classification of the SVM [18]. Several kind of kernels have been developed for SVM, for example, polynomial, dot product, and radial basis function (RBF) kernels. Among them, RBF kernel has shown to be more effective because it has good capacity to approximate nonlinear functions. Recently, wavelet kernel has been developed as an effective method for nonlinear approximation and mapping [4,19]. In [20], Zhang et al. has employed the wavelet kernel for the SVM classifier, and a wavelet SVM (WSVM) classifier has been proposed as a result. Since the wavelet transform provides better approximation capacity than the RBF, the WSVM classifier provides higher accuracy than the SVM with RBF kernel. Since then, the WSVM have been employed in many real applications, such as in the medical field [21], and machine fault diagnosis [22]. Due to the merits of the LSSVM classifier and the approximation capability of the wavelet kernel, a new least squares wavelet support vector machine (LSWSVM) is proposed first time in this paper to improve both computational efficiency and classification accuracy. However, the generalization performance of the LSWSVM is affected by its parameters. Thus, it is necessary to optimize the parameters to obtain a better performance. In the literature, Particle swarm optimization (PSO) [23] has been developed as an effective optimization technique to optimize parameters of a process. Compared with other optimization methods, PSO have many advantages, such as simple implementation, few parameters, parallel computation ability, and quickly converge [24]. The PSO had proved its optimization capacity when applying for many practical applications, such as for optimizing the parameters of SVMs [25] and other optimization problems [26,27]. Therefore, the PSO is used in this paper to effectively select the parameters of the LSWSVM, leading to a new PSO-LSWSVM classifier, which addresses all difficulties in the use of the SVM classifier.

In summary, the novelties and main contributions of this paper can be listed as follows:

- A new methodology for bearing fault diagnosis is developed by combining between feature extration based on a NLM-EMD method, a feature selection based on a mRMR and a new PSO-LSWSVM classifier.
- To improve the generalization performance of the SVM, a novel PSO-LSWSVM classifier, which combines between a least squares procedure, a new wavelet kernel function and the PSO, is proposed.

## 2. Feature Extraction

In this paper, we employ the NLM-EMD method, which has been developed in our previous work [5] and proved its effectiveness, to extract a rich bearing feature set. For the merit features of the NLM-EMD and its detail description, the interested readers can refer to the previous work [5].

### 2.1. Nonlocal Mean (NLM) De-Noising

Consider a noise signal has a form as y=u+n, where *u* is the true signal and *n* is an additive noise. The noise component can be eliminated using a NLM as below:

(1) $$\hat{u}\left(i\right)=\frac{1}{M(i)}\sum_{j\in \Omega_{i}}\omega \left(i,j\right)y\left(i\right)$$

The parameters used in (1) can be designed as in [5]. For more detail description of the NLM denoising, the interested readers can refer to our previous paper [5].

### 2.2. Empirical Mode Decomposition

Consider an original signal x(t), a number of IMFs C(t) can be obtained from the original signal using EMD method as [28]

(2) $$x\left(t\right)=\sum_{j=1}^{n}C_{j}\left(t\right)+r_{n}\left(t\right)$$

where the high frequency is decreased from $C_{1}\left(t\right)$, $C_{2}\left(t\right),\;C_{3}\left(t\right),\;\cdots ,\;C_{n}\left(t\right)$, and $r_{n}\left(t\right)$ contains no meaningful information. Generally, fault information is distributed significantly on the high and mid-frequency components [4,19]. Thus, the first five IMFs are used in this work for bearing fault analysis since they represent the mid- and high frequency components of the original signal.

### 2.3. Energy Feature Extraction

In the previous section, the EMD has been employed to decompose the original signal into a number of IMF components with different frequency bands. On the other hand, the frequency band can be referenced of the energy of fault vibration signal. Hence, in order to capture the effects of faults on the change of the energy of the vibration signal, IMF energy features are employed.

Each IMF component $C_{j}\left(t\right)$ possesses an energy $E_{j}\left(t\right)$, which can be calculated as:

(3) $$E_{j}=\int {\left|C_{j}\left(t\right)\right|}^{2}dt$$

Then, a normalization procedure can be applied for each $E_{j}\left(t\right)$:

(4) $$T_{j}=\frac{E_{j}}{T}$$

where *T* is the total energy of the first five IMF components:

(5) $$T={\left(\sum_{j=1}^{5}{\left|E_{j}\right|}^{2}\right)}^{\frac{1}{2}}$$

### 2.4. Time-Domain Feature Extraction

Time-domain features usually provide rich information to distinguish normal condition and fault condition. In this paper, the nine time-domain dimensionless parameters defined in Table 1 is used to extract fault information from the de-noised signal and the first five IMFs to obtain rich information of bearing faults.

Finally, a set of features, which includes 5+9×6=59 fetures, is obtained to represent a bearing condition.

## 3. Minimum Redundancy Maximum Relevance (MRMR) Feature Selection

Let *F* be the initial feature set and |S| be the cardinality in seeking feature subset *S*. The following criterion is developed for minimal redundancy:

(6) $$\min_{S\subset F}\frac{1}{{\left|S\right|}^{2}}\sum_{i,j\in \;S}I\left(f_{i},f_{j}\right)$$

and the maximum relevance criterion is defined as:

(7) $$\max_{S\subset F}\frac{1}{\left|S\right|}\sum_{i\in \;S}I\left(C,f_{i}\right)$$

where $I(f_{i},f_{j})$ is the mutual information of two features, $f_{i}$ and $f_{j}$; and $I(C,f_{i})$ quantifies the relevance of the feature, $f_{i}$, in *S* and the target class, *C*.

To obtain a feature subset with minimum redundancy and maximum relevance, a mRMR function is obtained by combining (6) and (7):

(8) $$\max_{S\subset F}\left(\sum_{i\in \;S}I\left(C,f_{i}\right)-\frac{1}{\left|S\right|}\sum_{i,j\in \;S}I\left(f_{i},f_{j}\right)\right)$$

The completed procedure of the mRMR can be refered to [4]. To obtain the desired feature subset, forward selection search [29] is employed.

## 4. PSO-LSWSVM

### 4.1. Least Squares Support Vector Machine (LSSVM)

Given a training set of *N* data points, $\left(x_{1},y_{1}\right),\;\left(x_{2},y_{2}\right),\;\ldots ,\;\left(x_{N},y_{N}\right)$, where $x_{i}\in \;R^{d}$ is the $i^{th}$ input vector and $y_{i}\in \;\pm 1$ is the corresponding target, we employ the idea of the transformation of an input pattern into a reproducing kernel Hilbert space using a set of mapping functions, ϕ(x). The reproducing kernel, $K(x,x^{\prime })$, in the reproducing kernel Hilbert space is the dot product of the mapping functions at *x* and $x^{\prime }$, i.e., $K\left(x,x^{\prime }\right)\;=\;\langle \phi \left(x\right).\phi \left(x^{\prime }\right)\rangle$. In the new defined kernel space, a linear classifier usually has a form below:

(9) y(x)=sign(ω.ϕ(x)+b)

To facilitate the selection of the parameters ω and *b*, the LSSVM formulates the optimization problem as:

(10) $$\begin{array}{l} \underset{\omega ,b,e}{\mathrm{minimize}}\;F(\omega ,b,e)=\frac{1}{2}\omega^{T}\omega +\frac{C}{2}\sum_{i=1}^{N}e_{i}^{2}\\ \mathrm{subject}\;\mathrm{to}\;y_{i}[\omega^{T}\phi (x_{i})+b]=1-e_{i} \end{array}$$

The feature mapping, i.e., ϕ(x), is usually unknown, and Mercer’s condition [30] can be appllied.

(11) $$\Omega_{ij}\;=\;y_{i}y_{j}\phi {\left(x_{i}\right)}^{T}\phi \left(x_{j}\right)$$

The decision function of the LSSVM classifier becomes:

(12) $$y_{i}\;=\;sign\left(\sum_{j=1}^{N}\alpha_{j}y_{j}K\left(x_{i},x_{j}\right)+b\right)$$

A kernel RBF can be chosen as:

(13) $$K\left(x_{i},x_{j}\right)\;=\;exp(-\frac{\left|\right|x_{i}-x_{j}{\left|\right|}^{2}}{\sigma^{2}})$$

where σ is a free parameter.

### 4.2. Least Squares Wavelet Support Vector Machine (LSWSVM)

Generally, the family of wavelet analysis has a form:

(14) $$h_{a,c}\left(z\right)\;=\;{\left|a\right|}^{-\frac{1}{2}}h(\frac{z-c}{a})$$

where z,a,c∈R, *a* is a dilation factor, *c* is a translation factor; and h(z) is the mother wavelet, which satisfies the following condition [31,32]:

(15) $$W_{h}\;=\;\int_{0}^{\infty }\frac{{\left|F\left(\omega \right)\right|}^{2}}{\left|\omega \right|}<\infty$$

where F(ω) is the output of h(z) using Fourier transform. Employing a wavelet transform for g(z), one obtains:

(16) $$W_{a,c}\left(g\right)\;=\;\langle g\left(z\right),h_{a,c}\left(z\right)\rangle$$

where 〈〉 indicates the dot product. The function g(z) is provided by [31]:

(17) $$g\left(z\right)\;=\;\frac{1}{W_{h}}\int_{-\infty }^{\infty }\int_{0}^{\infty }\frac{1}{a^{2}}W_{a,c}\left(g\right)h_{a,c}\left(z\right)dadc$$

Reformulate (17):

(18) $$\hat{g}\left(z\right)\;=\;\sum_{i=1}^{N}W_{i}h_{a_{i},c_{i}}\left(z\right)$$

where $W_{i}$ is the reconstruction coefficient, and g(z) is approximated by $\hat{g}\left(z\right)$.

A wavelet function can be selected as [31]:

(19) $$h\left(z\right)\;=\;\sum_{i=1}^{N}h\left(z_{i}\right)$$

where $z\;=\;{\left[z_{1},z_{2},\ldots ,z_{N}\right]}^{T}\in \;R$. Then, if $z,z^{\prime }\in \;R^{N}$, the dot-product wavelet kernels can be computed as:

(20) $$K\left(z,z^{\prime }\right)\;=\;\sum_{i=1}^{N}h\left(\frac{z_{i}-c_{i}}{a}\right)\sum_{i=1}^{N}h\left(\frac{z_{i}^{\prime }-c_{i}^{\prime }}{a}\right)$$

and the following expression is used to describe the translation invariant wavelet kernels [31]:

(21) $$K\left(z,z^{\prime }\right)\;=\;\prod_{i=1}^{N}h\left(\frac{z_{i}-z_{j}}{a}\right)$$

Substituting (21) into (12), the decision function of the LSWSVM classifier has a form below:

(22) $$y_{i}\;=\;sign(\sum_{j-1}^{N}\alpha_{j}y_{j}\prod_{j=1}^{N}h\left(\frac{x_{t,j}-x_{i,j}}{a_{i}}+b\right)$$

where $x_{t,j}$ and $x_{i,j}$ denote the $j^{th}$ element of $x_{t}$ and the $i^{th}$ training sample, $x_{i}$, respectively.

In order to approximate a general nonlinear model, in this paper, we propose to use the following wavelet kernel:

(23) $$h\left(x\right)\;=\;\lambda cos\left(k\frac{x}{a}\right).\exp\left(-\frac{x^{2}}{a^{2}}\right)$$

where *a* is a parameter of the RBF kernel; *k* and λ are new parameters that control the kernel shape. It is obvious to see from equation (23) that the performance of the defined wavelet kernel depends significantly on the selection of the parameters *a*, *k*, and λ. When the parameters *a*, *k*, and λ are changed, the shape of the kernel is changed. Therefore, it is needed to optimize these parameters to obtain a good performance of the system.

### 4.3. Particle Swarm Optimization (PSO) for Parameter Selection of LSWSVM—the PSO-LSWSVM Classifier

In order to get the optimal values of the parameters *a*, *k*, and λ, particle swarm optimization (PSO) [33] is employed in this paper. The detail description of the PSO can be referred to our previous work [4,19] to reduce the length of the paper. The velocity, position and the initial weight of the PSO are updated using the following three equations:

(24) $$v_{id}^{t+1}\;=\;\omega .v_{id}^{t}+c_{1}r_{1}.(p_{best,id}^{t}-x_{id}^{t}+c_{2}r_{2}.(g_{best,d}^{t}-x_{id}^{t}))$$

(25) $$x_{id}^{t+1}\;=\;x_{id}^{t}+v_{id}^{t+1}$$

(26) $$\omega_{k}\;=\;\omega_{max}-\frac{\omega_{max}-\omega_{min}}{iter_{max}}\times iter$$

The definitions of the parameters used in Equations (24)–(26) can be referred to [4,19].

The LSWSVM classification model constructed using the wavelet kernel function defined in (23) has four user-determined parameters, including a regularization parameter *C* and three kernel parameters, λ, *k* and *a*. In this paper, we use PSO to automatically select the parameters of the LSWSVM classifier; hence, a relatively new classifier, i.e., PSO-LSWSVM, is proposed. The step-by-step implementation details of parameters selection for the LSWSVM classifer based on PSO are described below.

*Step* *1:* Initializes the parameters of the PSO: the population *N*, the position and velocity of each particle (*C*, *a*, *k* and λ-parameters for LSWSVM).
*Step* *2:* Uses the following fitness function, which is obtained from the output of the LSWSVM classifier, to evaluate the initialized particles:
  (27) $$\mathrm{fitness}\;\mathrm{function}\;=\;\frac{N_{t}}{N_{t}+N_{f}}$$
  where $N_{t}$ and $N_{f}$ denotes the number of true and false classification, respectively.
*Step* *3:* Creates a new swarm by updating the velocity and position of each particle using (24) and (25).
*Step* *4:* For the new obtained swarm, the fitness values are computed and compared to update the $p_{best,i}$ and $G_{best}$ of the swarm.
*Step* *5:* Checks the termination condition: If the maximum number is reached, goes to Step 6. Otherwise, return to Step 3 and continue the closed-loop process.
*Step* *6:* Encodes the optimal parameter of the wavelet kernel of the LSWSVM classifier from the global best position, $G_{best}$.

## 5. Fault Diagnosis Methodology

**Figure 1:**
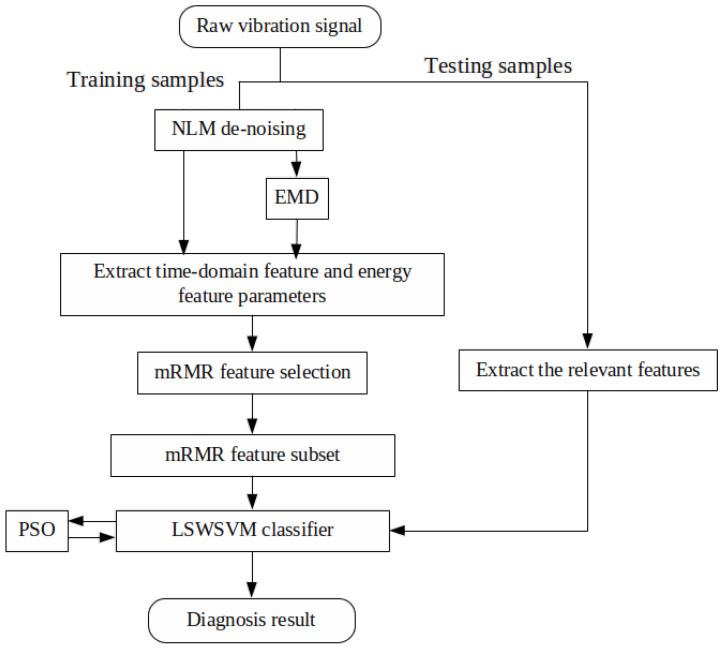
Flow chart of the developed strategy for bearing fault diagnosis.

**Figure 2:**
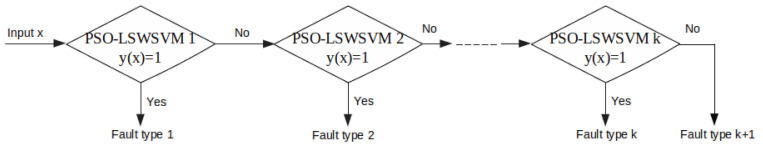
‘One to others’ multi-class fault classification system of PSO-LSWSVM.

The proposed fault diagnosis methodology is briefly described as in Figure 1. The implementation is executed as follows:

*Step* *1:* A number of effective IMFs are obtained after filtering the vibration signals using the NLM and EMD.
*Step* *2:* Extracts the energy and time domain features to obtain a combined feature set.
*Step* *3:* Uses the mRMR feature selection technique to get an optimal feature subset.
*Step* *4:* Uses the wavelet kernel function defined in (23) for LSSVM classifier and optimizes the parameters using the PSO technique.
*Step* *5:* Classifies the bearing fault types using the PSO-LSWSVM classifer based on the ‘one to others’ multi-class classification strategy [34], which is illustrated in Figure 2, and the selected feature subset in Step 3.

*Remark:* Although the full fault diagnosis system, which includes feature extraction, feature selection, and feature classification, is presented in this paper, the major contribution of this paper is to introduce a novel PSO-LSWSVM classifier. The feature extraction tasks are mainly taken from the previous work [4], while the feature selection based on the mRMR is a standard and well-known technique in the literature.

## 6. Experimental Results

### 6.1. Training and Test Data Configuration

**Figure 3:**
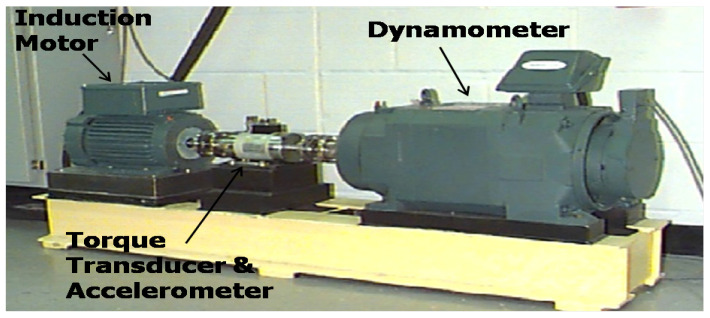
The bearing testbed

The data used in this experiment are taken from the Case Western Reserve University Bearing Data Center (2014) [35]. The bearing test-bed is shown in Figure 3. In this paper, four types of bearing conditions are considered, including one normal condition (no fault) which is labeled as NM and three fault conditions. The three fault conditions include fault at outer race, fault at inner race and fault at ball which are labeled as ORF, IRF and BF respectively. In each type of fault condition, fault size can have the value of 0.007, 0.014 or 0.021 mili-inches. Therefore, totally 10 conditions (10 classes) of bearing are taken into account.

### 6.2. Parameter Selection

**Figure 4:**
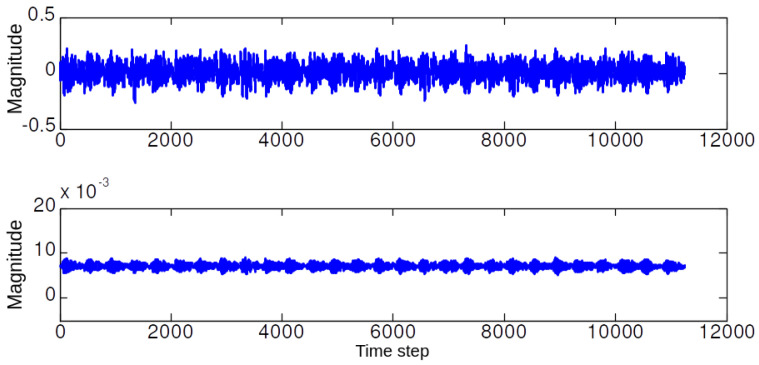
The represented (**top**) vibration signal, and (**bottom**) de-noised signal using NLM when the bearing in normal operation.

**Figure 5:**
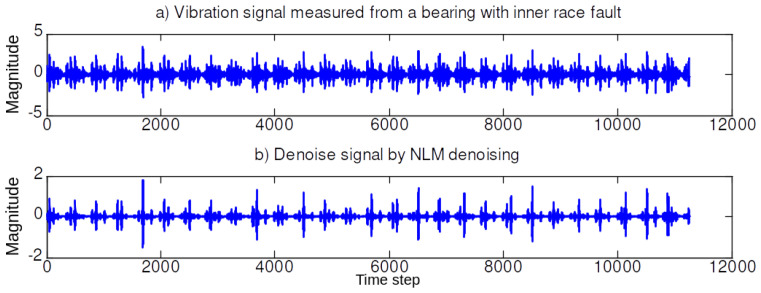
Denoising signal using NLM when the bearing in an inner race (IR) fault (0.021 in.).

**Figure 6:**
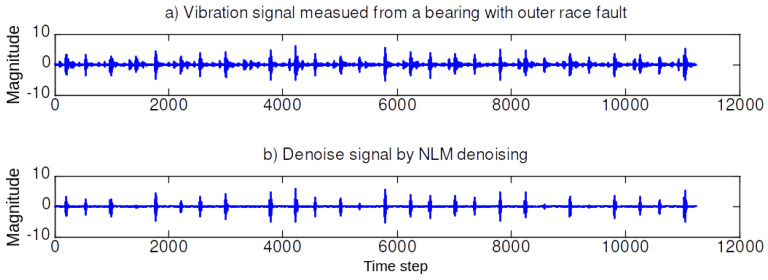
Denoising signal using NLM when the bearing in an outer race (OR) fault (0.021 in.).

**Figure 7:**
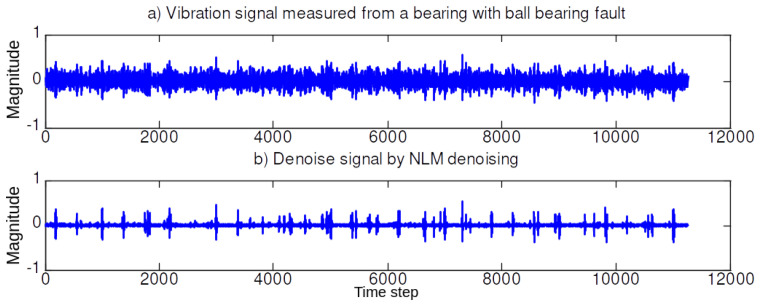
TDenoising signal using NLM when the bearing in a ball (B) fault (0.021 in.).

In the first simulation set, we illustrate the performance of the NLM and EMD. Figure 4, Figure 5, Figure 6 and Figure 7 illustrate the denoising results using the NLM. The denoised signals are then passed through the EMD to obtain the effective IMF components. The 59 features are then extracted from the denoised signal and the IMF components as described in Section 2.

In the second and third simulation sets, the computed feature set is fed into the mMRM feature selection to get an optimal feature subset. The selected feature subset is then used as input to a classifier to identify the bearing conditions. The LSWSVM classifier was implemented based on a modification of the LS-SVMLabtoolbox [36]. In order to verify the effectiveness of the PSO and the proposed wavelet kernel function, we constructed four different classifiers: (1) an LSRBFSVM classifier using an RBF kernel for the LSSVM with parameters selected by the user; (2) a PSO-LSRBFSVM classifier (LSRBFSVM with parameters are selected by using PSO); (3) an LSWSVM classifier using the proposed wavelet kernel in (25) with parameters selected by the users; and (4) a PSO-LSWSVM classifier (using PSO to automatically select the parameters of the LSWSVM). In addition, to verify the effects of the parameters λ, *k* and *a*, the PSO-LSWSVM classifier is used in three different circumstances: (**a**) λ and *k* are firstly selected by user, and the PSO is used to tune the parameters *a* and *C*; (**b**) λ is firstly selected, and the PSO is used to tune the parameters *k*, *a* and *C* simultaneously; and (**c**) the PSO is used to tune the parameters λ, *k*, *a* and *C* simultaneously. These classifiers are also compared with the k-nearest neighbor (KNN) [37] and probability neural network (PNN) [38] classifiers, which are widely applied for bearing fault diagnosis, to further verify the effectiveness of the proposed classifier.

### 6.3. Performance Evaluation

According to the forward selection search algorithm [29], 59 feature subsets are created based on the mRMR feature selection. To compare the generalization performance of the classifiers, we consider each feature subset as an independent dataset. Thus, we have 59 different datasets corresponding to 59 feature subsets. To evaluate the performance of the methods, the extracted feature vectors are used as inputs for the classifiers to obtain the classification accuracies. In this paper, to estimate the generalized classification accuracy, l-fold cross-validation (CV) [39], where *l* is set to 3, is employed. To obtain a precisely classification result, l-fold CV is performed ten times in this study.

#### 6.3.1. Training Process

First, the training process is performed to obtain an optimal feature subset of each classifier and the kernel parameters of the LSRBFSVM and LSWSVM classifiers. The PSO is performed at this training step. The validation accuracy in this study is computed as follows:

(28) $$C_{accuracy}\;=\;\frac{\sum_{K}N_{TP}}{N_{S}}\times 100\%$$

where K=10 indicates number of classes, $N_{TP}$ indicates the number of true classifications, and $N_{S}$ is the number of samples used in this experiment.

**Figure 8:**
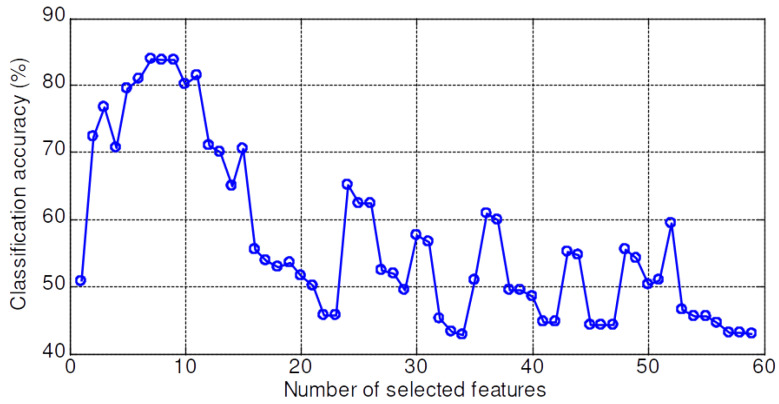
Validation accuracy of the KNN classifier.

**Figure 9:**
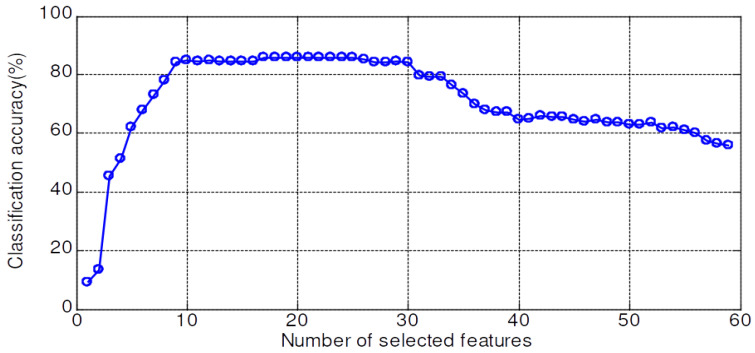
Validation accuracy of the PNN classifier.

**Figure 10:**
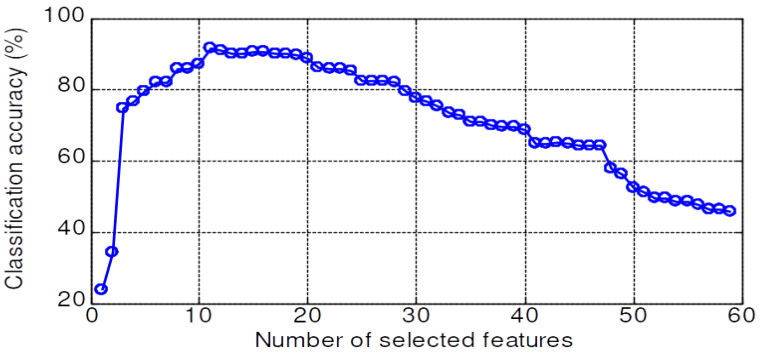
Validation accuracy of the LSRBFSVM classifier; C=4.5, σ=2.5.

**Figure 11:**
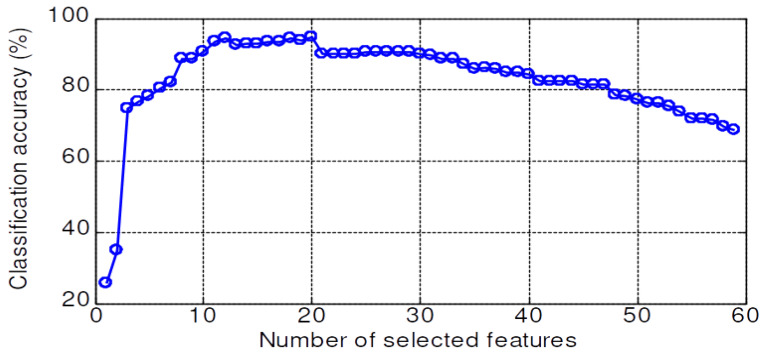
Validation accuracy of the PSO-LSRBFSVM classifier.

**Figure 12:**
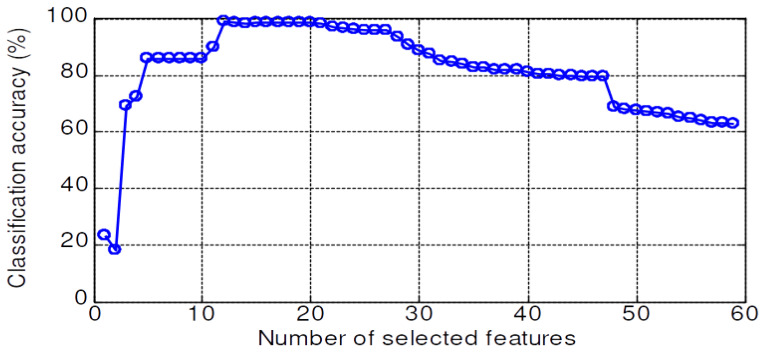
Validation accuracy of the LSWSVM classifier.

**Figure 13:**
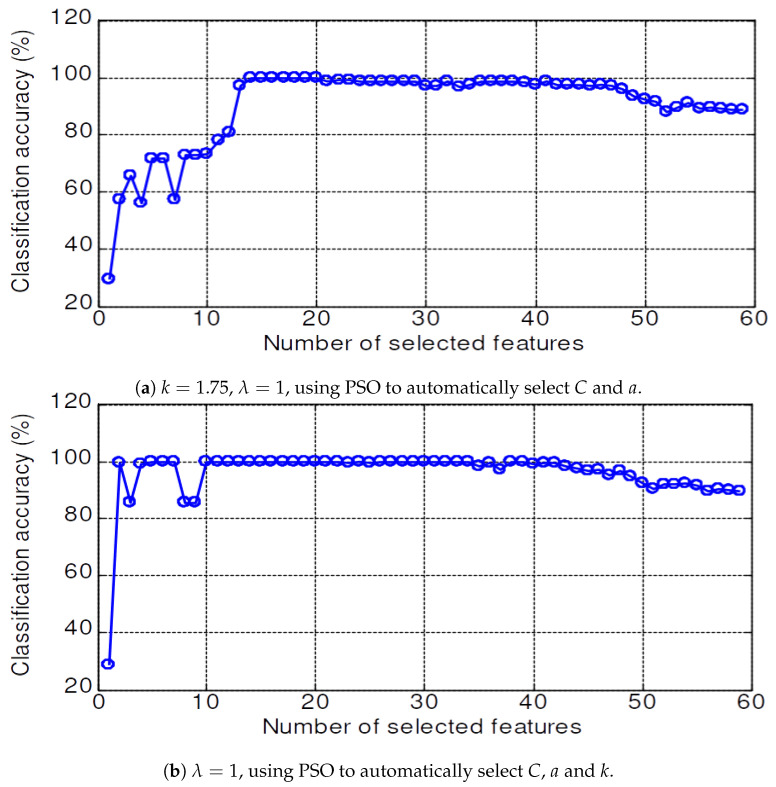

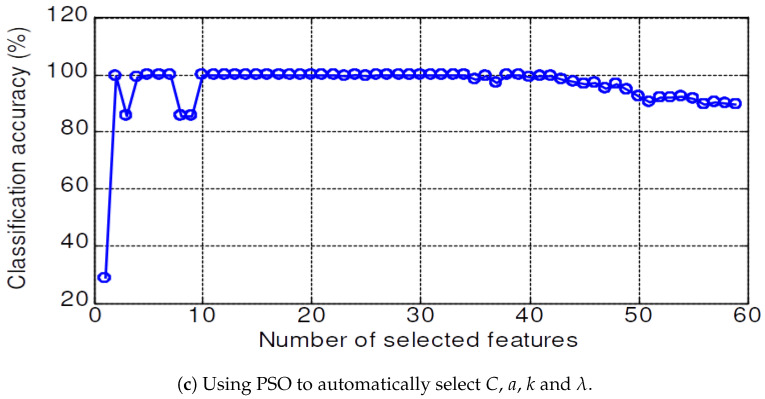
Validation accuracy of the PSO-LSWSVM classifier.

The validation accuracy of 59 features dataset for the KNN, PNN, LSRBFSVM, PSO-LSRBFSVM, LSWSVM, and PSO-LSWSVM classifiers are shown in Figure 8, Figure 9, Figure 10, Figure 11, Figure 12 and Figure 13, respectively. The mean and best results and the computational time (for one fold) of each method are also reported in Table 2 for the sake of comparison. The subspaces according to the best records are assigned as the optimal feature subset according to the forward selection search algorithm [29]. Observing from these figures, we can see that the combined 59 features yields a low classification accuracy due to the presence of the irrelevant and redundant features; for example, 43% for the KNN, 55.95% for the PNN, 45.71% for the LSRBFSVM, 68.57% for the PSO-LSRBFSVM, 62.86% for the LSWSVM, and around 90.95% for the PSO-LSWSVM. By using the mRMR criteria for feature selection, the classification accuracy is clearly increased. For example, for the KNN classifier, the peak value is obtained at 7 features with the accuracy increased up to 83.91%; for the PNN classifier, the peak value is obtained at 17 features with the accuracy increased up to 91.42%; for the LSRBFSVM, the peak value is obtained at 11 features with the accuracy increased up to 91.43%; for the PSO-LSRBFSVM, the peak value is obtained at 20 features with the accuracy increased up to 94.76%; for the LSWSVM, the peak value is obtained at 12 features with the accuracy increased up 99.05%; and for the PSO-LSWSVM, the peak value is obtained at 2 features with the accuracy increased up to 100%.

From these results, four observations can be obtained: (1) the feature subsets selected by the mMRM commonly yield higher accuracy than the use of all 59 features; (2) although the computational time of the PSO-LSWSVM (PSO: 30.52 s + LSWSVM: 0.422 s) classifier is higher than the KNN (0.125 s), PNN (0.109 s) and the PSO-LSRBFSVM classifier (PSO: 24.49 s + LSRBFSVM: 0.375 s), it gives much better performance. It should be notice that although the PSO requires a higher computational time, however the PSO training is done offline, and thus it will not affect to the real time fault diagnosis; (3) comparison results between Figure 12 with Figure 8, Figure 9 and Figure 10 shown that the LSWSVM classifier provides better accuracy compared to the KNN, PNN and LSRBFSVM classifiers; (4) by comparing Figure 11 with Figure 10 and Figure 13 with Figure 12, it is clear that using the PSO for parameters selection always provides better performance than using the random selection. In addition, comparisons between Figure 13a–c shown that all parameters, λ, *k*, *a* and *C*, have significant effects on the performance of the LSWSVM classifier, and that the selection of four parameters simultaneously will produce better generalization performance. Based on Table 2 and the forward selection search algorithm [29], 8 features, 17 features, 20 features and 2 features are selected as the optimal feature subset for the KNN, PNN, PSO-LSRBFSVM and PSO-LSWSVM classifiers, respectively.

#### 6.3.2. Testing Process

After the optimal feature subset and optimal model are selected for each classifier, the testing data samples are used to verify the effectiveness of the classifiers. The confusion matrices that show the performances of the KNN, PNN, PSO-LSRBFSVM and PSO-LSWSVM (using PSO to automatically select all parameters, i.e., λ, *k*, *a* and *C*) classifiers are shown in Table 3, Table 4, Table 5 and Table 6, respectively. From the results, it is obvious to see that the proposed PSO-LSWSVM classifier (accuracy = 95.33%) gives superior classification accuracy compared to the KNN (accuracy = 83.05%), PNN (accuracy = 84.77%), and PSO-LSRBFSVM (accuracy = 86.84%).

## 7. Conclusions

Two major contributions have been presented in this paper:

- A new pattern recognition approach for bearing fault diagnosis is developed by combining between feature extration based on a NLM-EMD method, a feature selection based on a mRMR and a new PSO-LSWSVM classifier.
- A novel PSO-LSWSVM classifier, which combines between a least squares procedure, a new wavelet kernel function and the PSO, is proposed.

In the presented method, the combined NLM-EMD is first employed to acquire more effective IMF components of vibration signals. Then, for the de-noised signal and each IMF component, the energy and time-domain feature parameters are extracted to obtain characteristic parameters. Next, the mRMR feature selection technique is adopted to eliminate the irrelevant and redundant features and select the best combined feature subset. Finally, the selected feature subset is fed into the proposed PSO-LSWSVM classifier to identify the bearing conditions, wherein a novel combination of a PSO, a least squares procedure, and a new wavelet kernel is proposed to address the difficulties in the use of the traditional SVM classifier. By experimenting with a real bearing vibration signal, we verified that the proposed wavelet kernel function has a better generalization performance than the previous kernels, i.e., RBF kernel, and the proposed PSO-LSWSVM classifier can overcome all difficulties in the use of the traditional SVM classifer. In addition, the uses of the NLM-EMD for the feature extraction and mRMR for the feature selection are effective. Therefore the proposed fault diagnosis methodology based on the NLM-EMD, mMRM feature selection and PSO-LSWSVM classifier improves the bearing recognition accuracy significantly, up to 95.53%.

## Figures and Tables

**Table 1:** Time-domain dimensionless parameters.

No.	Feature	Equation	No.	Feature	Equation
1	Standard deviation	$x_{std}=\sqrt{\frac{\sum_{n=1}^{N}{\left(x\left(n\right)-x_{m}\right)}^{2}}{N}}$	6	Root mean square	$x_{rms}=\sqrt{\frac{\sum_{n=1}^{N}{\left(x\left(n\right)\right)}^{2}}{N}}$
2	Peak	$x_{p}=max\left|x\left(n\right)\right|$	7	Clearance factor	$CLF=\frac{x_{p}}{{\left(\frac{1}{N}\sum_{n=1}^{N}\left|x\left(n\right)\right|\right)}^{2}}$
3	Skewness	$x_{skew}=\frac{\sum_{n=1}^{N}{\left(x\left(n\right)-x_{m}\right)}^{3}}{\left(N-1\right)x_{std}^{3}}$	8	Shape factor	$SF=\frac{x_{rms}}{\frac{1}{N}\sum_{n=1}^{N}\left|x\left(n\right)\right|}$
4	Kurtosis	$x_{kur}=\frac{\sum_{n=1}^{N}{\left(x\left(n\right)-x_{m}\right)}^{4}}{\left(N-1\right)x_{std}^{4}}$	9	Impulse factor	$IF=\frac{x_{p}}{\frac{1}{N}\sum_{n=1}^{N}\left|x\left(n\right)\right|}$
5	Crest factor	$CF=\frac{x_{p}}{x_{rms}}$	where x(n) is a signal series for n=1,2,…,N.		

**Table 2:** Accuracy comparison (%) among classifiers.

Classifier	Mean	Max	Position	Computation Time (s)
kNN	56.83	83.91	7	0.125
PNN	70.92	85.95	17	0.109
LSRBFSVM	71.37	91.42	11	0.375
PSO-LSRBFSVM	82.80	94.76	20	24.49
LSWSVM	81.40	99.05	12	0.422
PSO-LSWSVM (a)	90.15	100	14	30.52
PSO-LSWSVM (b)	95.97	100	5	30.52
PSO-LSWSVM (c)	98.14	100	2	30.52

**Table 3:** Confusion matrix for showing classification results of the KNN classifier.

	NM	ORF1	IRF1	BF1	ORF2	IRF2	BF2	ORF3	IRF3	BF3
NM	1452	4	6	0	10	1	11	2	3	1
ORF1	8	889	10	2	15	4	57	15	50	0
IRF1	0	0	1126	2	10	342	12	8	7	7
BF1	0	3	10	1135	5	3	0	7	10	0
ORF2	2	1	4	0	1249	2	13	73	1	24
IRF2	0	0	299	0	2	1144	3	5	2	3
BF2	38	191	2	0	11	0	1270	0	28	0
ORF3	0	20	2	18	192	1	1	1346	22	0
IRF3	0	7	41	12	6	3	3	4	1377	0
BF3	0	385	0	331	0	0	130	40	0	1465
Sensitivity(%)	96.8	59.27	75.07	75.67	83.27	76.27	84.67	90.07	91.8	97.67
Specificity(%)	99.72	98.85	97.1	99.73	99.13	97.64	98.02	98.07	99.45	93.47
Accuracy (%)	83.05									

**Table 4:** Confusion matrix for showing classification results of the PNN classifier.

	NM	ORF1	IRF1	BF1	ORF2	IRF2	BF2	ORF3	IRF3	BF3
NM	1485	2	18	45	2	5	10	0	3	7
ORF1	0	1304	43	55	4	6	27	3	5	21
IRF1	1	53	1053	1	5	305	14	0	415	3
BF1	0	59	18	1290	10	2	33	7	16	9
ORF2	0	0	5	100	1250	1	42	65	0	0
IRF2	0	0	299	0	5	1177	0	0	3	1
BF2	14	13	0	0	101	0	1337	0	0	8
ORF3	0	0	0	3	63	0	0	1325	0	13
IRF3	0	2	64	6	60	4	0	50	1058	2
BF3	0	67	0	0	0	0	37	50	0	1436
Sensitivity(%)	99.0	86.93	70.2	86.0	83.33	78.47	89.13	88.33	70.53	95.73
Specificity(%)	99.32	98.79	94.5	98.86	98.42	97.72	99.99	99.41	99.45	98.86
Accuracy (%)	84.77									

**Table 5:** Confusion matrix for showing classification results of the PSO-LSRBFSVM classifier.

	NM	ORF1	IRF1	BF1	ORF2	IRF2	BF2	ORF3	IRF3	BF3
NM	1483	2	7	1	1	2	5	0	1	3
ORF1	0	1227	60	0	7	4	61	4	9	14
IRF1	1	20	1005	55	25	270	20	1	179	0
BF1	0	20	1	1276	3	0	0	0	2	5
ORF2	0	15	8	0	1354	0	41	62	1	0
IRF2	0	0	342	0	1	1215	5	0	0	1
BF2	15	78	0	1	0	0	1320	1	2	30
ORF3	0	50	0	0	80	0	0	1408	5	9
IRF3	0	31	77	97	28	9	0	1	1301	1
BF3	1	57	0	70	1	0	48	23	0	1437
Sensitivity(%)	98.87	81.80	67.00	85.07	90.27	81.00	88.00	93.87	86.73	95.80
Specificity(%)	99.84	98.82	95.93	99.77	99.06	97.42	99.06	98.93	99.45	98.52
Accuracy (%)	86.84									

**Table 6:** Confusion matrix for showing classification results of the PSO-LSWSVM classifier.

	NM	ORF1	IRF1	BF1	ORF2	IRF2	BF2	ORF3	IRF3	BF3
NM	1500	0	2	0	1	0	5	0	0	2
ORF1	0	1484	0	0	1	0	1	0	2	0
IRF1	0	0	1210	0	10	164	0	0	60	0
BF1	0	0	25	1493	1	0	2	0	0	4
ORF2	0	0	20	0	1464	0	3	31	0	0
IRF2	0	0	178	0	2	1317	5	0	8	3
BF2	0	12	0	1	1	18	1473	1	0	0
ORF3	0	0	0	1	20	0	0	1447	0	0
IRF3	0	0	65	0	0	1	0	21	1421	0
BF3	0	4	0	5	0	0	11	0	9	1491
Sensitivity(%)	100	98.93	99.43	80.67	97.60	87.80	98.20	96.47	94.73	99.40
Specificity(%)	99.93	99.97	99.92	98.27	99.60	98.55	99.76	99.84	99.45	99.79
Accuracy (%)	95.33									

## Abbreviations

List of Abbreviation used in paper:

BF	Ball falut bearing
EMD	Empirical mode decomposition
IMF	Intrinsic mode function
IRF	Inner race fault bearing
KNN	K-nearest neighbor
PNN	Probability neural network
PSO	Particle swarm optimization
LSSVM	Least squares support vector machine
LSWSVM	Least squares wavelet support vector machine
LSRBFSVM	Least squares radial basis function support vector machine
mRMR	Minimum-redundancy maximum-relevance
NLM	Nonlocal mean
NM	Normal bearing
ORF	Outer race fault bearing
RBF	Radial basis function
SMO	Sequential minimal optimization
SVM	Support vector machine
WSVM	Wavelet support vector machine
